# Vascular Inflammation in Lungs of Patients with Fatal Coronavirus Disease 2019 (COVID-19): Possible Role for the NLRP3 Inflammasome

**DOI:** 10.21203/rs.3.rs-842167/v1

**Published:** 2021-09-01

**Authors:** Oindrila Paul, Jian Qin Tao, Eric West, Leslie Litzky, Michael Feldman, Kathleen Montone, Chamith Rajapakse, Christian Bermudez, Shampa Chatterjee

**Affiliations:** University of Pennsylvania Perelman School of Medicine; University of Pennsylvania Perelman School of Medicine; University of Pennsylvania Perelman School of Medicine; University of Pennsylvania Perelman School of Medicine; University of Pennsylvania Perelman School of Medicine; University of Pennsylvania Perelman School of Medicine; University of Pennsylvania Perelman School of Medicine; University of Pennsylvania Perelman School of Medicine; Penn: University of Pennsylvania

**Keywords:** Hyperinflammation, SARS-CoV-2, acute respiratory disease syndrome (ARDS), fatal COVID-19, NLRP3

## Abstract

**Background:**

Hyperinflammation is a key event that occurs with SARS-CoV-2 infection. In the lung, hyperinflammation leads to structural damage to tissue. To date, numerous lung histological studies have shown extensive alveolar damage, but there is scarce documentation of vascular inflammation in postmortem lung tissue.

**Methods:**

Lung sections from 8 COVID-19 affected and 11 non-COVID-19 subjects [of which 8 were acute respiratory disease syndrome (ARDS) affected and 3 were from subjects with non-respiratory diseases] were stained for H & E to ascertain histopathological features including presence of thrombi/microthrombi. Inflammation along the vessel wall was also monitored by quantification of the expression of moieties of the NLRP3 inflammasome pathway (NLRP3 and caspase-1).

**Results:**

In lungs from “fatal COVID-19”, vascular changes in the form of microthrombi in small vessels, arterial thrombosis, and organization were extensive as compared to lungs from “non-COVID-19 non respiratory disease” affected subjects. The NLRP3 pathway components were significantly higher in lungs from COVID-19 subjects as compared to non-COVID-19 fatal cases without respiratory disease. No significant differences were observed between COVID-19 lungs and non-COVID-19 ARDS lungs.

**Conclusion:**

We posit that inflammasome formation along the vessel wall is a characteristic of lung inflammation that accompanies COVID-19. Thus, the NLRP3 inflammasome pathway seems to be probable candidate that drives amplification of inflammation post SARS-CoV-2 infection.

## Introduction

It has been more than a year since the pandemic caused by the novel SARS-CoV-2 corona virus (Severe Acute Respiratory Syndrome Coronavirus), also known as COVID-19 has affected large populations globally [1, 2]. The virus disproportionately affects the respiratory system and a major cause of fatality is the acute respiratory distress syndrome (ARDS) that accompanies the infection [3, 4]. Autopsy-based lung histological studies have been an invaluable tool in understanding the pathobiology of COVID-19; indeed these have shown indications of inflammation, edema, coagulopathy and fibrosis [3, 5–8]. COVID-19 manifests itself under a wide spectrum of symptoms, but it can broadly be classified as an inflammatory disease where excessive inflammation is the main driver of poor clinical outcome [9, 10]. In this direction, the vascular endothelium, a dynamically adaptable interface that is actively involved in recruitment of inflammatory cells, possibly plays a crucial role in regulation, progression, and amplification of inflammation. While post mortem findings have shown alveolar damage, early or intermediate proliferative phase, and presence of thrombi and signs of inflammation in the lungs [3, 6, 8], histopathology in the context of vascular inflammation and altered vascular structures has been somewhat scarce [7, 11].

Inflammatory processes involve the participation of inflammasomes that are multimeric platforms assembled in response to pathogenic stimuli. Dysregulated inflammasome signaling has been well established as a pivotal event in hyper-inflammatory syndromes [12–14]. Among the inflammasomes, the NLRP3 inflammasome comprising of the NLRP3 subunit, ASC and caspase-1, is well established to be activated in response to microbial infection [15, 16] and to drive cell death [17, 18]. It is also involved in COVID-19, as evidenced by the detection of inflammasome subunits and products in the sera and lung tissue of COVID-19 patients [19, 20]. However, there are no reports of the presence of the inflammasome in the pulmonary vasculature with COVID-19 infection. As the vasculature seems to be crucial in inflammation accompanying COVID-19, the status of NLRP3 along the vascular wall needs to be documented.

We posit that inflammasome formation is characteristic of pulmonary vascular inflammation that accompanies COVID-19. The purpose of this study is to contextualize vascular features in lung tissue in fatal cases of COVID-19 as compared to other pulmonary diseases and ascertain NLRP3 expression along the vascular wall. Here we document the major histological findings of 8 postmortem examinations done on patients with clinically confirmed COVID-19 and compare these to lungs of non-COVID-19 subjects. This study contributes to the growing data on this topic [3, 6, 21–24].

## Materials And Methods

We analyzed lung tissue samples of 8 patients that died of COVID-19 in 2020 and 11 patients that died from non-COVID complications. Written informed consent was obtained for postmortem examination from the next of kin of these patients. For the COVID-19 patients, SARS-CoV-2 infection was confirmed by real time PCR analysis at the time of hospital admission. Autopsies were done by trained personnel using personal protective equipment in accordance with the recommendations of the University of Pennsylvania School Of Medicine.

Tissue blocks taken from the most representative areas of the lung (as identified by macroscopic examination) were fixed in formalin. Paraffin embedded sections of 3 to 5 μm thickness were stained with hematoxylin and eosin (H & E). Images were captured on the Aperio Pathology System and visualized by ImageScope (Leica Biosystems, Buffalo Grove, IL). High and low powered fields were selected for evaluation. Inflammation and inflammation induced cell death (pyroptosis) were characterized by immunostaining for NLRP3 inflammasome and caspase-1 respectively. Sections were deparaffinized; after antibody retrieval, were stained using anti-human NLRP3 monoclonal antibody at 1:200 or anti-human caspase antibody at 1:100 (both from R&D Systems, Minneapolis, MN). Secondary antibody used was conjugated to Alexa 488 at 1:200 (Life Technologies, Eugene, OR). Appropriate IgG controls were used to fix exposure settings. Vectashield antifade mounting medium used was from Vector Labs (Burlingame, CA). Images were acquired by epifluorescence microscopy using a Nikon TMD epifluorescence microscope, equipped a Hamamatsu ORCA-100 digital camera, and MetaMorph imaging software (Universal Imaging, West Chester, PA, USA). Fluorescence images were acquired at excitation = 488 nm; all images were acquired with the same exposure and acquisition settings as reported previously [25–27]. Quantitation of the fluorescence signal was carried out using the MetaMorph Imaging Software. Integrated Intensities were normalized to the field area as reported by us elsewhere ^40^.

## Results

Patient demographics and clinical information are summarized in Tables 1 and 2, histological characteristics in Tables 3 and 4. COVID-19 patients were 4 men and 4 women, with a mean age of 71.8 years (SD 13.9); non-COVID-19 patients were 7 men and 4 women, with a mean age of 64 (SD 10.7). Lung sections from all patients showed diffuse alveolar damage including hyaline membranes, intra-alveolar fibrin deposition, and thickening of the alveolar-capillary membrane. All sections from lungs also stained positively for the NLRP3 inflammasome associated markers that were assessed and quantitated by fluorescence imaging.

**Figure 1:**
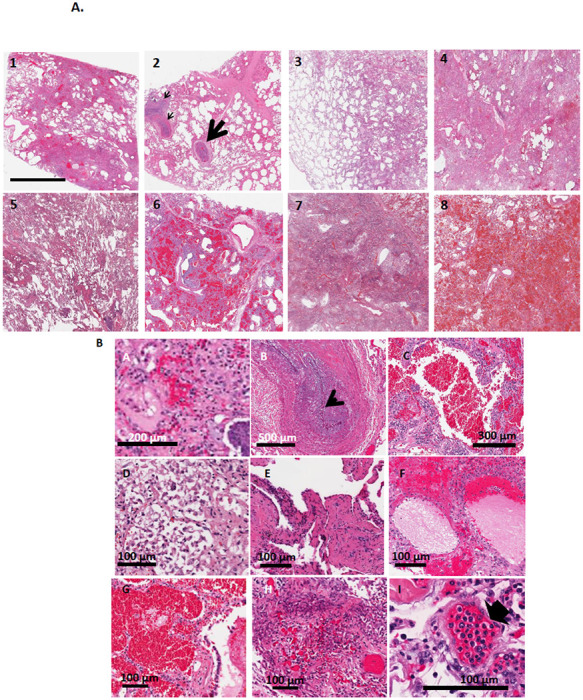
Hematoxylin and Eosin-stained sections staining from representative regions of the lung parenchyma of post-mortem lung tissue of 8 COVID-19 patients. A. All patients show extensive alteration of lung microstructure in the form of alveolar damage, fibrin exudation into alveolar space, thrombi and fibroblastic proliferation. The septa are thickened and there is presence of hyaline membranes and dense infiltrates. Scale bar is 3 mm. 1: Alveolar damage with collagen deposition and exudative pattern of damage 2. Large thrombi and smaller caliber arteries showing fibrin thrombi (arrows) 3. Alveolar damage pattern arising from fibroblastic proliferations 4 and 5. Exudate in the entire lung 6. Necrosis with blood and exudate in the lung parenchyma 7. Hemorrhagic infarction of lung tissue adjacent to a pulmonary artery with thrombotic material 8. Pulmonary hemorrhage with blood and fibrin exudation into the parenchyma B. H and E staining at higher magnification: All patients had extensive diffuse alveolar damage, microthrombi and edema in regions of the lung. A. Fibroblastic proliferation B. Plugged airway due to remodeling C. Coagulation necrosis with blood in the lung tissue D. Proliferative phase of diffuse alveolar damage E. Patchy distribution of damage F. Proteinaceous exudates in alveolar spaces G. Blood and fibrin exudation into parenchyma H: Fibroblastic proliferation I: Endotheliitis of small vessel <100 μm with infiltration of the vessel wall by lymphocytes (arrow shows infiltrated cells) C. (unavailable with this version):Thrombi and microthrombi were identified in 7 of the 8 patients. Images of vessels were chosen to emphasize the microthombi. Box is magnified in the right panel. Arrow shows microthombi on alveolar septa.

Upon light microscopic examination, the lungs of all COVID-19 patients showed extensive alteration of lung microstructure (Fig. 1A, B). A closer inspection of COVID-19 lungs revealed fibrin exudation into alveolar space, extensive thrombi and fibroblastic proliferation, hyaline membrane, fibrin deposition and early and advanced proliferative phase of diffuse alveolar damage (Fig. 1B). Thrombi and microthrombi were identified in 7 of the 8 patients (Fig. 1C). Vascular changes were extensive, with microthrombi in small vessels and arterial thrombosis and organization. Microthrombi were also observed in alveolar septa. Thrombi and microthrombi were found in >75–80% of the fields imaged. Histological findings are detailed in the legends of Fig. 1 and in Table 2.

**Figure 2:**
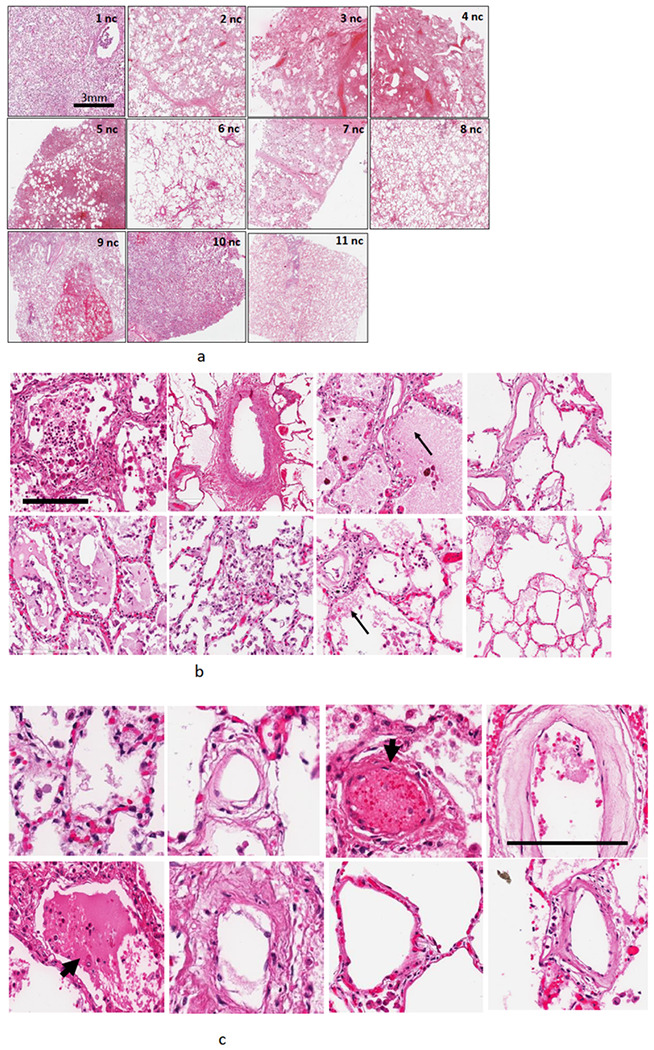
A. Hematoxylin and Eosin-stained sections staining from representative regions of the lung parenchyma of post-mortem lung tissue of 11 non COVID-19 patients. Scale bar is 3 mm. B. H and E staining at higher magnification: All patients had diffuse alveolar damage, microthrombi and edema in regions of the lung. Arrows show proteinaceous exudate in the airspaces. Scale bar is 200 microns C. Vascular structures in lungs from non-COVID-19 sources. Arrows show thrombi in vessels. About 40% of the fields showed thrombi. Scale bar is 100 microns.

In contrast, the lungs from non-COVID fatal cases, showed less thrombi and fibrin exudation (Fig. 2A, B). While higher magnification showed certain key features of lung injury such as diffuse alveolar damage, thickening of the alveolar-capillary membrane, fibroblastic proliferation, the presence of hyaline membranes, edema and proliferative phase of diffuse alveolar damage, the non-ARDS lungs (nc 1, 8 and 11) have intact structure and did not show alveolar infiltration or hemorrhage (Fig. 2B). Furthermore, in non-COVID-19 lungs, vessels showed thrombus in about < 40% of the fields (Fig. 2C).

**Figure 3:**
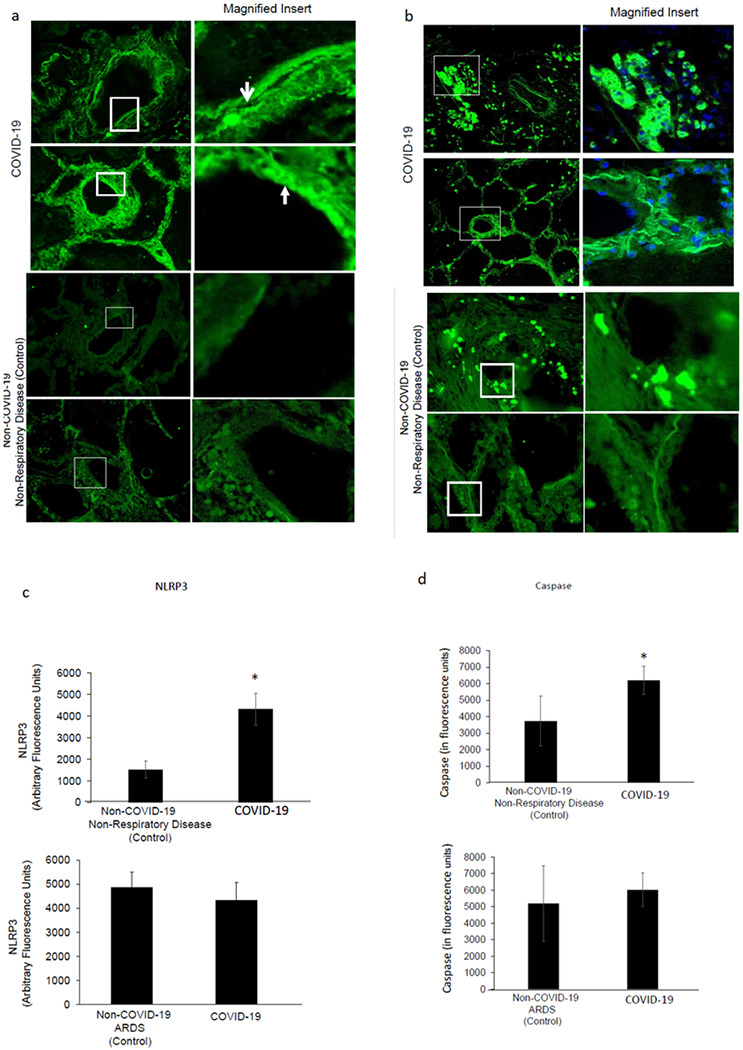
Inflammasome in the lungs of patients with COVID-19 infection. Representative images of the immunofluorescence in lung sections stained with anti-NLRP3 and Caspase-1. A. The NLRP3 subunit (green) was visualized along the walls of arterioles (arrow). Upper panels: COVID-19 lungs. Lower Panels: Lungs from non-COVID 19 subjects, without respiratory disease. B. Caspase staining (green). Upper panels: COVID-19 lungs. Lower Panels: Lungs from non-COVID 19 subjects, without respiratory disease. C and D. Quantitation of the fluorescence Intensity of the images using MetaMorph Imaging Program. *p<0.01 as compared to non-COVID lungs.

We next assessed the expression of the NLRP3 subunit and its downstream effector caspase-1 in all samples. In lungs from COVID-19 subjects, intense expression of the NLRP3 and caspase-1 as observed from the green-fluorescent signal, is shown in Fig. 3. Fluorescence around the vessel walls implied NLRP3 expression along the endothelial layer (Fig. 3A, upper panels). The effector enzyme, caspase-1 was widely distributed throughout the lungs and was not limited to the vascular structures (Fig. 3B, upper panels). In lungs from non-COVID subjects that were not affected by respiratory disease (nc1, 8 and 11), NLRP3 (Fig. 3A, lower panels) and caspase-1 expressions were significantly lower (Figs. 3A, B: lower panels and Fig. 3C). However, in lungs of subjects, that were affected by ARDS, NLRP3 and caspase1 expression was not significantly different from COVID-19 lungs (Fig. 3D).

## Discussion

COVID-19 has been described largely as a respiratory disease; indeed, the respiratory tract and alveolus are amongst the primary sites of infection. However, it is also an inflammatory disease where release of inflammatory cytokines is the cause of organ injury and damage. The endothelium is the converging site of the inflammation as its activation (expression of adhesion molecules and cytokines) leads to immune cell recruitment; thus it is reasonable to conclude that COVID-19 is potentially a vascular disease [11, 28, 29]. While this would be an indirect impact of the virus, more recent studies also provide evidence of a direct effect i.e. infection by SARS-CoV-2 virus of endothelial cells [30]. Our inspection of autopsies of the 8 COVID-19 patients showed macro and microthrombi in almost all fields imaged, indicating coagulation pathology. This was not observed in autopsies of non-COVID-19 lung sections. As is well established, coagulation is closely linked to endothelial inflammation signaling; inflammatory moieties on the endothelium increase leukocyte infiltration and alter coagulation control driving a procoagulant direction [31]. Thus, COVID-19 which is increasingly being described as a vascular disease should perhaps be more accurately defined as a pathology which has its origins in “endothelial inflammation” signaling.

Inflammasome activation on the endothelium plays a major part in cell death and injury with inflammation. The NLRP3 inflammasome is a multiprotein complex comprised of three basic components: (1) A sensor such as a NOD-like receptor (NLR) (2) the adaptor protein apoptosis-associated speck-like protein containing a caspase-recruitment domain (ASC) and (3) the inflammatory cysteine aspartase caspase-1. The assembly of this complex leads to release of caspase-1 which then exerts its catalytic activity on the pro-inflammatory cytokines (IL-1β) that after their release perpetuate cell death, specifically inflammation induced cell death or pyroptosis [17, 18].

A recent report showed high levels of NLRP3 inflammasome and caspase-1 in patients with fatal COVID [20]. This is not surprising as increased NLRP3 is associated with various inflammatory lung pathologies including acute lung injury and ARDS [32, 33]. The COVID-19 lung autopsies in this study, showed NLRP3 expression throughout the lung, but intense expression was seen along the lung vessel walls implying inflammasome expression on the endothelium. The downstream effector of NLRP3 inflammasome activation, Caspase-1 was found to be expressed throughout the lungs including in the vascular structures. Caspase-1 is considered as a key pyroptotic mediator; it reportedly drives pulmonary vascular endothelial cell death [17]. Elsewhere too, high caspase-1 expression has been reported with both COVID-19 [20] and with other lung inflammatory pathologies [34]; however its expression on the endothelium or vascular wall with COVID-19 has not been documented. Possibly the NLRP3-caspase-1 axis can directly (via caspase-1 driven pyroptosis) or indirectly (via NLRP3 driven chemotactic immune cell recruitment [35]) injure the endothelial layer. This confluence of vascular injury, thrombosis and dysregulated inflammation seems to propagate lung damage with COVID-19 and supports a pivotal role for the pulmonary endothelium in severe and fatal COVID-19. In contrast, non-COVID-19 lungs of subjects that did not have respiratory disease, had significantly lower expression of NLRP3 and caspase-1, indicating that an engagement of the NLRP3 pathway in COVID-19 and in ARDS.

As NLRP3 inflammasome driven pyroptosis is being considered to play a leading role in the pathogenesis of multi-organ failure with COVID-19 [36], there is some speculation on the mechanisms by which inflammasome activation occurs upon SARS-CoV-2 infection. One possibility is that the SARS-CoV-2 spike protein’s binding to cell surface-expressed angiotensin-converting enzyme 2 (ACE2) directly triggers its enzymatic activation and alters membrane polarity that can result in activation of NLPR3 inflammasome [37]. Or NLRP3 could be activated via Angiotensin II which is reported to facilitate the assembly of the inflammasome. A third possibility could be via interaction of damage associated molecular patterns (DAMPs that are released post infection) and members of the complement cascade with the SARS-CoV-2 virus. Potent cleavage fragments of DAMPs and complement cascade can potentially activate the inflammasome [38]. Yet another possibility is that the stretch from ventilation activates the inflammasome [39]. Once activated around the vascular wall (endothelial layer), the NLRP3 inflammasome would lead to release of caspase-1 and interleukin-1β that would facilitate pyroptosis (cell death) of the endothelium (Schema 1).

To the best of our knowledge, this is the first study on NLRP3 expression in the vascular structures in lungs of fatal cases of COVID-19. The origin of several events that exacerbate inflammation and injury with COVID-19 (such as immune cell aggregation and extravasation, edema, formation of thrombi and leukopenia) possibly lies in pulmonary endothelial inflammasome activation and pyroptotic cell death. Therefore, NLRP3 inhibitors have been suggested for as a potential treatment strategy and are currently being explored for management of moderate COVID-19 symptoms (NCT04540120) [19, 40].

A major drawback of this study is that our sample size is small. Moreover, paraffin based post-mortem samples offer a snapshot of the disease and cannot recreate the evolving disease process. Histology is also impacted with the effects of clinical care and treatment as comorbidities, ventilation and medication pose as challenges in interpretation of results. Nevertheless, this study identifies endothelial NLRP3 inflammation, and documents thrombi and altered vascular structures in the lungs of fatal COVID-19 patients.

## Conclusions

Taken together, our data show that in COVID-19 affected subjects, lungs show inflammasome formation in the specifically along the vessel wall. This indicates a role for NLRP3 inflammasome pathway in amplification of inflammation post SARS-CoV-2 infection and a potential usage of antagonists or blockers of the NLRP3 pathway in COVID-19 inflammation regulation and control. Overall, this report adds to the growing list of studies on COVID-19 associated pulmonary pathology that highlight the importance of vascular endothelial inflammation in progression to severe and fatal disease.

## Figures and Tables

**Figure 4:**
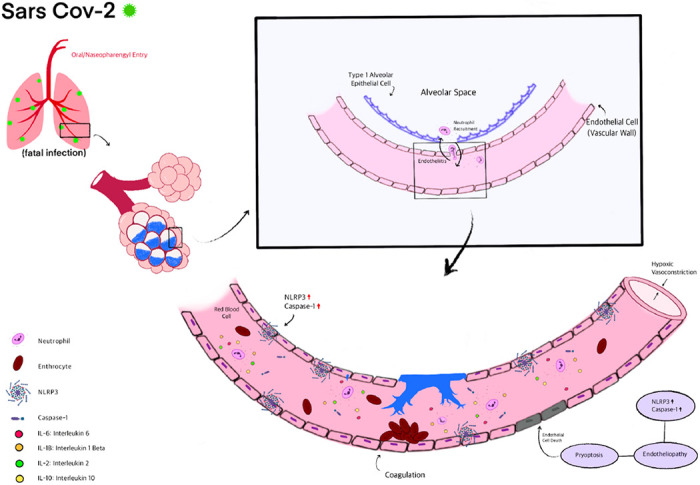
Overview of SARS-CoV-2 entry, infection and endothelial inflammation and cell death. As is well established, oral nasopharyngeal entry of SARS-CoV-2 is followed by its binding to the alveolar epithelium. The infected pneumocytes secrete cytokine and chemokines, which attract neutrophils to the alveolar space, leading to a possible breach of the alveolar wall. Meanwhile, endothelial cells overexpress NLRP3 as we observed in the autopsies (either by infection, or via increased amounts of chemokines and cytokines). The NLRP3 pathway drives endothelial pyroptosis. The leads to breakdown of the endothelial-alveolar barrier and causes interstitial and alveolar space flooding. Endothelial cell death and debris activates coagulation cascades that promotes thrombi formation.

**Table 1:** Patient characteristics, comorbidities, select immunostaining findings on a score of 0 to 3: 0, absent; 1, mild; 2, moderate; 3, severe.

Patient	Gender	Age	Known Medical History	Substance Abuse (Smoking/Alcohol)	Thrombi/microthombi	NLRP3 expression	NLRP3 activation (caspase-1)
1.	Female	61	Asthma and Stroke	Non-smoking	2	2	3
2.	Female	63	Breast cancer and therapy related Acute Leukemia	Smoking	3	2	3
3.	Female	73	COPD	Smoking and Alcohol	3	2	3
4.	Female	94	COPD, Coronary Artery Disease and Sjogrens disease	Not known	3	2	3
5.	Male	50	Myeloproliferative disorder and Pulmonary/portal Hypertension	Not known	3	2	3
6.	Male	72	Dementia, Diabetes and Hypertension	Not known	3	3	3
7.	Male	77	Pulmonary Embolism and Deep Vein Thrombosis and Hypertension	Not known	3	3	3
8.	Male	85	Cerebral Vascular Disease	Not known	3	2	3

**Table 2:** Patient characteristics, comorbidities, select immunostaining findings for non-COVID-19 lungs on a score of 0 to 3: 0, absent; 1, mild; 2, moderate; 3, severe.

Patient	Gender	Age	Known Medical History	Substance Abuse (Smoking/Alcohol)	Thrombi/microthombi	NLRP3 expression	NLRP3 activation (caspase-1)
1 nc.	Male	60s	Heart Transplant	Not Known	1	2	1
2 nc.	Female	50s	Emphysema	Not Known	1	3	2
3 nc.	Male	80s	Emphysema	Not Known	1	3	3
4 nc.	Male	40s	Bronchopneumonia	Not Known	2	3	3
5 nc.	Female	60s	Diffuse alveolar damage; chronic lung disease	Not Known	1	2	2
6 nc.	Male	70s	End stage lung disease	Not Known	1	2	3
7 nc.	Female	60s	Diffuse alveolar damage; COPD and renal cell carcinoma	Not Known	2	3	3
8 nc.	Female	50s	Mild edema in lung	Not Known	1	1	1
9 nc.	Male	70s	COPD	Not Known	1	3	3
10nc.	Male	70s	Aspiration pneumonia; diabetes	Not Known	2	2	2
11nc.	Male	50s	Sarcoid	Not Known	1	1	1

**Table 3:** Pulmonary pathological features in COVID-19 autopsy cases on a score of 0 to 4: 0, absent; 1, mild 2, moderate; 3, high; 4, severe.

Patient No.	Hyaline Membranes	Interstitial Fibrosis	Atypical pneumocytes	Pulmonary hemorrhage	Morphological aspects
1.	4	4	4	3	Proliferative phase of diffuse alveolar damage, thrombi/microthrombi.
2.	3	3	4	3	Emphysematous change, microthrombi, alveolar septal thickening, thrombi/microthrombi.
3.	4	4	4	3	Pulmonary edema, alveolar septal thickening
4.	4	4	4	4	Proliferative phase of diffuse alveolar damage, pulmonary hemorrhage, thrombi/microthrombi.
5.	4	4	4	3	Diffuse alveolar damage, Advanced proliferative phase, thrombi/microthrombi.
6.	4	4	4	4	Advanced proliferative phase, pulmonary hemorrhage, thrombi/microthrombi.
7.	4	4	4	4	Exudative phase diffuse alveolar damage, hemorrhage, thrombi/microthrombi.
8.	4	4	4	4	Advanced proliferative phase, hemorrhage, thrombi/microthrombi.

**Table 4:** Pulmonary pathological features in non-COVID-19 (nc) autopsy cases on a score of 0 to 4: 0, absent; 1, mild 2, moderate; 3, high; 4, severe.

	Hyaline Membranes	Interstitial Fibrosis	Atypical pneumocytes	Pulmonary hemorrhage	Morphological aspects
1 nc.	2	3	2	2	Fibrotic pattern, hyaline membranes,
2 nc.	3	3	4	3	Intracapillary hyaline thrombi
3 nc	4	4	4	1	Fibrosis, hyperplasia, proteinaceous exudate in alveoli.
4 nc	4	4	4	3	Acute fibrinous and organizing pneumonia, Microthrombi, hyaline membranes
5 nc	4	4	4	4	intra-alveolar edema, hemorrhage, capillary congestion, proteinaceous hyaline membrane
6 nc	4	4	4	3	Observed vascular changes in the form of diffuse (micro)vascular damage with large thrombi
7 nc	4	4	4	2	Proteinaceous exudate in alveolar space, hyaline membranes.
8 nc	1	1	1	1	Mild alveolar distention
9 nc	3	2	2	3	Extensive presence of fibrin within the alveolar spaces, proteinaceous exudate
10 nc	4	4	4	4	Exudative phase of diffuse alveolar damage
11 nc	2	2	2	1	Presence of hyaline membranes, fibrin aggregates

## Data Availability

The samples, datasets and analysis of this study are available from the corresponding author on reasonable request.

## Abbreviations

ACE2: Angiotensin-converting enzyme 2
ARDS: Acute Respiratory Distress Syndrome
ASC: Apoptosis-associated speck-like protein containing a caspase-recruitment domain
COVID-19: Coronavirus disease of 2019.
H & E: Hematoxylin and Eosin
NLRP3: NOD-like receptor protein 3
SARS-CoV-2: Severe Acute Respiratory Syndrome Coronavirus
